# A hepatoprotective experiment on taro vegetable (*Colocasia esculenta* (L.) Schott) flower employing animal models by mitigating oxidative stress

**DOI:** 10.1002/ame2.70031

**Published:** 2025-05-28

**Authors:** Mahathir Mohammad, Fahmida Tasnim Richi, Rabiul Hossain, Md. Arafat, Pair Ahmed Jiko, Nazim Uddin Emon, Sayed Al Hossain Rabbi, Tirtha Khastagir, Md. Hemayet Hossain, Safaet Alam

**Affiliations:** ^1^ Department of Chemistry Chittagong University of Engineering & Technology Chittagong Bangladesh; ^2^ Department of Pharmaceutical Chemistry, Faculty of Pharmacy University of Dhaka Dhaka Bangladesh; ^3^ Department of Pharmacy University of Asia Pacific Dhaka Bangladesh; ^4^ Department of Pharmacy University of Science and Technology Chittagong Bangladesh; ^5^ Department of Biochemistry and Biotechnology University of Science and Technology Chittagong Chittagong Bangladesh; ^6^ Department of Pharmacy, Faculty of Science and Engineering International Islamic University Chittagong Chittagong Bangladesh; ^7^ Department of Chemistry Government City College, Chattogram, National University Chattogram Bangladesh; ^8^ Department of Pathology Marine City Medical College Chattogram Bangladesh; ^9^ Chemical Research Division, BCSIR Dhaka Laboratories Bangladesh Council of Scientific and Industrial Research (BCSIR) Dhaka Bangladesh

**Keywords:** animal models, antioxidant, *Colocasia esculenta*, hepatoprotective, histopathology, taro vegetable

## Abstract

**Background:**

*Colocasia esculenta* (L.) Schott, known as the taro vegetable, possesses various beneficial effects and is traditionally used in folk medicine. This study explores the ameliorative antioxidant and hepatoprotective effect of a methanolic extract of the *C. esculenta* flower (ME‐CEF) against oxidative damage and hepatotoxicity in mice.

**Methods:**

The antioxidant efficacy of ME‐CEF was assessed using 2,2′‐azino‐bis‐(3‐ethylbenzothiazoline‐6‐sulfonic) (ABTS) and 2,2‐diphenyl‐1‐picrylhydrazyl (DPPH) scavenging assay. The hepatoprotective effect was investigated by an assessment of liver injury indicators (amino transferase [ALT], aspartate amino transferase [AST], alkaline phosphatase [ALP], bilirubin, creatinine) and normalizing lipid profiles (cholesterol [CHO], triglyceride [TG], high‐density lipoprotein [HDL], and low‐density lipoprotein [LDL]) along with histopathological study and antioxidant enzymes (CAT). A phytochemical analysis, both qualitative and quantitative, was conducted, including gas chromatography‐tandem mass spectrometry (GC–MS/MS) analysis and an in silico molecular docking study.

**Results:**

The Result Showed that ME‐CEF Possesses Moderate ABTS and DPPH Scavenging Activity with IC_50_ Values of 117.18 and 160.41 μg/mL. As Illustrated by Reducing Liver Enzymes (ALT, AST, ALP, Bilirubin, Creatinine) and Lipid Profile (CHO, TG, LDL) and Raising HDL Levels (*p* < 0.01), ME‐CEF Dose Dependently Mitigated CCl_4_ ‐Induced Acute Liver Injury. Furthermore, ME‐CEF Blocked Hepatic Oxidative Stress by Boosting Antioxidant Enzymes (CAT) and Preventing Liver Tissue Damage and Apoptosis. In Silico Investigations Also Showed a Promising Binding Affinity with Tumor Necrosis Factor α (TNF‐α), Interleukin 6 (IL‐6), PRAP‐1, and Xanthin Oxidoreductase, which Displayed Antioxidant and Hepatoprotective Candidacy while Notable Safety and Efficacy Profile Was Also Documented through ADME/T Studies. Histopathological Analysis Showed Reduced Hepatocellular Necrosis and Vascular Congestion in Silymarin and Extract Groups.

**Conclusion:**

Based on these results, our findings strongly recommend the medicinal use of the plant, highlighting its antioxidant and hepatoprotective potentials.

## Graphical abstract

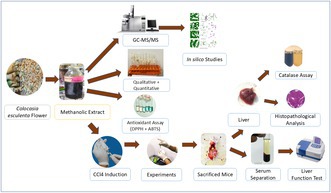

1. The antioxidant efficacy of *Colocasia esculenta* flower’s methanolic extract was assessed using 2,2′‐azino‐bis‐(3‐ethylbenzothiazoline‐6‐sulfonic) (ABTS) and 2,2‐diphenyl‐1‐picrylhydrazyl (DPPH) scavenging assay.
2. The hepatoprotective effect was investigated by assessing liver injury indicators (amino transferase [ALT], aspartate amino transferase [AST], alkaline phosphatase [ALP], bilirubin, creatinine) and normalizing lipid profiles (cholesterol [CHO], triglyceride [TG], low‐density lipoprotein [LDL], and high‐density lipoprotein [HDL]) along with histopathological study and antioxidant enzymes (CAT).
3. A qualitative and quantitative phytochemical analysis was performed, including GC–MS/MS analysis and an in silico molecular docking study.

## 1 INTRODUCTION

The liver is a large organ that carries out complex functions like metabolism, detoxification, and other vital processes. It is made up of two main types of cells: hepatocytes (the main working cells) and biliary cells (supporting cells).^1^ The liver performs biotransformation and metabolism of xenobiotics, which can lead to the deterioration of parenchymal cells and liver injury. Xenobiotics are lipophilic in nature.^2^ Halogenated alkane CCl_4_ is an organic xenobiotic. It causes reversible acute liver injury at high doses.^3^ It builds up in the hepatocyte's endoplasmic reticulum. CYP450 enzymes break it down and produce free trichloromethyl radicals (CCl_3_), and these react with O_2_ to produce trichloromethyl‐peroxyl radical (CCl_3_O_2_) and reactive oxygen species (ROS).^4^, ^5^ Free radicals are unpaired, unstable, highly reactive free electrons. There are two types of free radicals: oxygen‐based (ROS) and nitrogen‐based (reactive nitrogen species [RNS]) that result in various diseases (inflammation, cancer, aging, arthritis, liver fibrosis).^6^ Excessive ROS levels (hydroxyl radicals and hydroxyl peroxide) can lead to binding intracellular lipids or proteins; inactivate enzymes form lipid peroxidation and oxidative damage and lead to cell death due to necrosis and apoptotic mechanism and the expulsion of inflammatory cytokines (interleukin 1β [IL‐1β], tumor necrosis factor α [TNF‐α], G protein–coupled receptor), consequently leading to liver damage.^7^ Moreover, the injured liver can release cytosolic enzymes (AST, ALP, AST, bilirubin) into the blood.^8^

Natural products (NPs) include a diverse range of bioactive substances used in human and veterinary medicine and agriculture. Some medications in use or under clinical trials for antifungal, antibacterial, anticancer, and hypercholesterolemia are derived from the synthetic or semi‐synthetic modification of natural compounds.^9^ From 2005 to 2007, 13 medications derived from natural sources had been authorized, notably the initial members of novel pharmacological classes: the peptides exenatide and ziconotide, and the small molecules ixabepilone, retapamulin, and trabectedin. These drugs cover various therapeutic areas and exhibit diverse chemical structures.^10^

*Colocasia esculenta* (taro) is used as an edible vegetable plant for its leaves, corm, and flower and is grown in tropical regions as it belongs to the *Araceae* family. It is indigenous to Southeast Asia and Africa. It exhibits dark green coloration on its upper portion and light green on the underside and is primarily found in wet regions.^11^ Eating raw tuber and leaves is poisonous due to the presence of calcium oxalate. Leaves contain anthocyanins that protect against liver damage and helminthic disease.^12^ Leaf juice is used as rubefacient and purgative.^13^ Corm juice is used as a diuretic, demulcent, and anodynes agent.^14^ Taro contains antioxidants and bioactive compounds, including carotenoids and is used to treat cancer. *C. esculenta* also possesses potent antimicrobial,^15^ antidiabetic,^16^ and antimetastatic properties.^17^ Taro contains 97% vitamin A, which aids the eye, and its high vitamin C content supports immunity.^18^ In the presence of saponin and alkaloids, *C. esculenta* leaves exhibits hepatoprotective property.^19^ However, scientific studies on the hepatoprotective activity against *C. esculenta* flower remain inconclusive. Conventional treatment does not recover liver injury and causes side effects. We highlight the need for alternative medicine due to the vast mortality rates among liver patients. Thus, using CCl_4_‐induced oxidative stress and liver injury, we investigate the possible hepatoprotective effect of *C. esculenta* flower.

## 2 MATERIALS AND METHODS

### 2.1 Chemicals

Methanol, chloroform, olive oil, and CCl_4_ were purchased from a local scientific store. Silymarin was purchased from Radiant Pharmaceutical Limited. Normal saline and olive oil were purchased from a regional medicine store. Folin–ciocaltu reagent, 2,2‐diphenyl‐1‐picrylhydrazyl (DPPH), 2,2′‐azino‐bis‐(3‐ethylbenzothiazoline‐6‐sulfonic) (ABTS), and other solvents were provided by Merck and were purchased from a local supplier.

### 2.2 Plant collection and preparation

Fully grown *C. esculenta* flowers were harvested in September 2023 from Feni District, Bangladesh. A botanist and professor at the University of Chittagong, named Dr. Shaikh Bokhtear Uddin, verified the plant's validity (accession no.: CE/CU/2056/mm). After the flowers were thoroughly cleaned under running water, any unnecessary or rotten particles were removed, and their spadices were separated, the flowers were sliced into tiny fragments and allowed to dry for 4 weeks at room temperature (between 28°C and 32°C). Subsequently, the dehydrated flowers were ground using an electric blender and stored in a secure, tightly sealed container until the extraction procedure.

### 2.3 Preparation of extract

An amount of 100 g of the finely powdered dried sample was added to 400 mL of methanol to extract the *C. esculenta* flower powder. The extraction process involved soaking the mixture for 3 days at room temperature, stirring constantly on the first day. The extraction was filtered using vacuum filtration, filter paper, and clean cotton. The extraction technique was completed twice by adding 400 mL of methanol to the sample residue each time. Each extraction's residue was incorporated and allowed to dry using a rotary evaporator at ambient temperature under the fan. The final extract was preserved in the refrigerator with a glass vial.

### 2.4 Extraction yield

The extraction yields were calculated by the following equation:

$$\%\text{Yield}=\frac{\text{Extract weight after evaporating}}{\mathrm{Dry}\;\text{weight of powder}}\times 100.$$

### 2.5 Experimental animals

In the experiments conducted, adult Swiss albino mice were used. Mice weighing approximately 30–35 g were sourced from the Animal Research Division of the Bangladesh Council of Scientific and Industrial Research (BCSIR) in Chittagong. These mice were housed under standard environmental conditions, a temperature of 25°C ± 2°C and 12 h of light–dark cycle, with humidity levels at 55%–60% for acclimatization at the University of Science and Technology Chittagong (USTC) animal facility. After this period, the mice were transferred to regular polypropylene cages, ensuring consistent access to food and water throughout the study. Ethical approval was obtained from the Department of Pharmacy at the USTC in Bangladesh, adhering to guidelines established by the Institutional Animal Ethics Committee of the Faculty of Biological Science at USTC under ethical clearance number USTMEBBC/23/7/27.

### 2.6 Preparation of administration doses

The in vitro antioxidant experiment was conducted using three doses: 250, 500, 1000 μg/mL. The dose levels for the plant extract were 200 mg/kg body weight (BW) and 400 mg/kg BW for the in vivo antioxidant and hepatoprotective tests, respectively. Additionally, CCl_4_, standard, and control were used. As the Organization for Economic Co‐Operation and Development (OECD) (2008) directed, normal saline was used to vehicle and prepare the methanolic extract's in vivo dosage. The serial dilution technique was prepared in vitro dosages from stock solutions. Ascorbic acid served as the reference drug, and all medications were meticulously prepared at each step of the acute oral toxicity trials, adhering to OECD criteria.^20^

### 2.7 Acute oral toxicity

Swiss albino mice (30–35 g) of either sex (*n* = 21) were used for the toxicity research done according to the standard defined by the OECD (2008).^21^ Mice were starved for 16 h and randomly divided into seven groups of three. Each mouse in the control group received distilled water (2 mL/kg orally), whereas test groups were supplied varied doses of the extract (200, 400, 800, 1000, 2000, and 5000 mg/kg p.o.) to identify safe levels. After they received enough nutrition and hydration, the mice were observed for 1 h, then often for 4 h, and again for 24 h to monitor behavioral, autonomic, and neurological responses. The observation was continued for 14 days to determine the mortality rates.^22^

### 2.8 Qualitative analysis

The entire ME‐CEF was qualitatively analyzed for the presence of chemical components. Qualitative phytochemical assessment for the detection of alkaloids, carbohydrates, flavonoids, steroids, tannin, glycoside, and saponin was performed for the extract using the previously presented method.^23^

### 2.9 Quantitative analysis

#### 2.9.1 Total phenolic content

Total phenolic content (TPC) was evaluated using the folin–ciocalteu reagent (FCR) as an oxidizing agent. The polyphenolic contents in the test sample take on a blue hue when reduced by FCR. A ultraviolet (UV) spectrophotometer was used to measure the absorbance of the extract at 650 nm after it had been combined with diluted FCR and Na_2_CO_3_, which were then incubated in the dark. Gallic acid was used to draw a standard curve and determine the TPC, which is measured in milligrams of gallic acid equivalents (GAE) per gram. The same FCR reduction technique was used to create a standard calibration curve by plotting concentration against absorbance for a given quantity of gallic acid.^24^

$$Y=0.0019x+0.0135;R^{2}=0.9984$$

where *x* is the gallic acid equivalent (mg/g), and *Y* is the absorbance.

#### 2.9.2 Total flavonoid content

The aluminum chloride technique was used to determine the extract's total flavonoid content (TFC).^23^ Four milliliters of distilled water were mixed with 50 μL of the diluted crude extract (1 mg/mL) and 3 mL of 5% NaNO_2_ solution. After a 5‐min incubation period, 0.3 mL of a 10% AlCl_3_ solution was added, and the mixture was allowed to settle for 6 min. After that, 2 mL of 1 M NaOH was added, and double‐distilled water was used to bring the volume down to 10 mL. Using pure water as a blank, absorbance was measured at 510 nm during a 15‐min incubation period at room temperature. A quercetin calibration curve was used to calculate the flavonoid concentration of each analysis, which was carried out in triplicate.

$$Y=0.0013x+0.0451,R^{2}=0.9971$$

where *Y* is the absorbance, and *x* is the quercetin equivalent (mg/g).

#### 2.9.3 Total tannin content

The FCR was used to measure the tannins. Specifically, 50 μL of the sample extract was mixed with 950 μL of distilled water, 500 μL of FCR, 2.5 mL of a 35% NaCO_3_ solution, and the mixture was diluted to 10 mL with purified water. The mixture was thoroughly mixed and allowed to sit at room temperature for 40 min. At 725 nm, absorbance was measured using water as a blank instead of a sample. The equation for tannin acid equivalent was derived from a standard tannic acid calibration curve.^24^

$$Y=0.21x+0.0135;R^{2}=0.9998$$

where *Y* is the absorbance, and *x* is the tannic acid equivalent (mg/g).

### 2.10 GC–MS/MS analysis

Therapeutic chemicals from CEF‐ME were examined using the electron impact ionization (EI) approach using a mass spectrometer (GC–MS/MS TQ 8040, Shimadzu, Kyoto, Japan) linked to a gas chromatograph (GC–MS/MS, Shimadzu, Japan). The following temperature settings were used with a fused silica capillary column (Rxi‐30 m, 0.25 mm ID, 0.25 m, 5 ms): the column was heated to 500°C for 1 min, 200°C for 2 min, and 300°C for 7 min after commencing at 50°C. The gas chromatography system ran at a pressure of 53.5 kPa, with a total flow rate of 11.0 mL/min and a column flow rate of 1.0 mL/min. The 39‐min GC–MS/MS analysis collected data in the 50–600 *m/z* range using the Q3 scan mode. The relative abundance in qualitative GC–MS/MS analysis was ascertained by contrasting each compound's peak area with the total or prominent peak. Identification is made easier with this procedure using relative numbers and fragmentation patterns.

### 2.11 In vitro antioxidant activity

#### 2.11.1 2,2′‐Azino‐bis‐(3‐ethylbenzothiazoline‐6‐sulfonic) assay

The method employed for testing was described previously by Floegel et al.^25^ First, ABTS was dissolved in 524.62 g/mol distilled water with 2.45 mM potassium persulfate (K_2_S_2_O_8_), then it was left in the dark for 12–16 h at 37°C. Subsequently, the solution was diluted with distilled water to achieve an absorbance of approximately 0.7 Å at 734 nm. After this, 100 μL of extract at varying concentrations (250, 500, and 1000 μg/mL), obtained through serial dilution from the stock solution, was mixed with 3.9 mL of ABTS solution and incubated for 30 min before measuring the absorbance at 734 nm. All test samples were repeated thrice. The formula was used to employ (%) scavenging capability.

$$\begin{array}{c} \;\left(\%\right)\;\text{Scavenging}=\frac{\text{Absorbance of}\;\left(\text{control}-\text{sample}\right)}{\text{Absorbance of control}}\times 100 \end{array}$$

#### 2.11.2 2,2‐Diphenyl‐1‐picrylhydrazyl assay

According to Shah and Modi,^26^ the DPPH method was used to analyze the antioxidant activity. The DPPH solution was prepared in 4‐mg DPPH mixed with 100‐mL methanol; various concentrations of the extracts (250, 500, and 1000 μg/mL) were subsequently added to 3.9‐mL of the DPPH solution. The absorbance changes were measured at 517 nm. Ascorbic acid was used as standard. These measurements were performed in triplicate, and (%) inhibition was calculated using the following equation:

$$\left(\%\right)\;\text{Inhibition}=\frac{\text{Absorbance of}\;\left(\text{control}-\text{sample}\right)}{\text{Absorbance of control}}\times 100$$

### 2.12 Hepatoprotective activity

#### 2.12.1 In vivo experimental design

To address acute liver injury, a 50% solution of CCl_4_ by a mixture of 1:1 CCl_4_ and olive oil was administered to mice. A total of 25 selected Swiss albino mice were selected for the experiment. Among these, 20 mice received CCl_4_ intraperitoneally at a dose of 1 mL/kg BW (1:1 CCl_4_ in olive oil) for 7 days, and 5 mice were separated for the control group. After 24 h, those mice were divided into five groups.

Group 1: control group: received normal saline at a dose of 1 mL/kg p.o. once daily
Group 2: CCl_4_ group: received CCl_4_ at a dose of 1 mL/kg i.p. once for 7 days
Group 3: CCl_4_ + standard group: received standard drug silymarin 100 mg/kg p.o. once daily
Groups 4 and 5: treated group: CCl_4_ + ME‐CEF 200 and CCl_4_ + 400 mg/kg BW p.o. once daily

After 7 days of treatment, an anesthesia overdose (Ketamine HCl (100 mg/kg) and Xylazine (7.5 mg/kg)) through the intraperitoneal route was given to the mice models followed by euthanasia. Blood was taken by heart punching and collected in a gel tube. The blood samples were centrifuged to remove the serum and were used for the evaluation of biochemicals. The mice's livers were removed; a portion of the liver was preserved in 10% formalin for histopathological analysis, and the remaining portion was collected for the assessment of oxidative stress markers.^27^

#### 2.12.2 Lipidic profile

The lipidic profile was determined by quantifying plasmatic levels of cholesterol (CHO), triglyceride (TG), high‐density lipoprotein (HDL), and low‐density lipoprotein (LDL) using an autoanalyzer machine (Mindray BS 230).

#### 2.12.3 Biochemical marker analysis

The serum biochemical analysis uses liver function tests from each treated group such as alanine amino transferase (ALT), aspartate amino transferase (AST), alkaline phosphatase (ALP), and bilirubin. The renal function test creatinine was analyzed using a commercially available autoanalyzer machine (Mindray BS 230).

#### 2.12.4 Oxidative stress indicator

##### Catalase activity

Standard protocols described by Aebi,^28^ with some modifications, were used to measure catalase activity in liver tissue samples, which involved measuring the degradation of hydrogen peroxide (H_2_O_2_) substrate by catalase collected from animal models. A 1 g of tissue was homogenized in 0.05 M of 10 mL of phosphate buffer (pH 7.0) and centrifuged at 10000 rpm for 10 min at 4°C for the study of catalase activity. 500 μL of 0.34 mM H_2_O_2_, 2.5 mL of 0.05 M phosphate buffer, and 40 μL of supernatant were added after 10‐min measured absorbance at 240 nm.

$$\%\text{Inhibition}=\frac{\text{Absorbance of}\;\left(\text{control}-\text{treated}\right)}{\text{Absorbance of control}}\times 100$$

#### 2.12.5 Histopathological studies

Livers from the experimental mice were excised and cut into small pieces. These samples were fixed overnight in a 10% formaldehyde solution followed by dehydration. The dehydrated tissues were then embedded in paraffin. Using a microtome, 4‐μm sections were sliced. The liver sections were dewaxed in xylene, rehydrated through a series of alcohol grades and washed with distilled water for 5 min. They were stained with the basic dye hematoxylin for 40 s and counterstained with the acidic dye eosin for 20 s. After staining, the slides were examined under an OLYMPUS CX43 microscope at 40× and 100× magnification to identify damage, including vascular changes, vacuolar degeneration, loss of cellular structure, and alterations in cell architecture. Architectural changes were particularly noted in the CCl4‐induced group. Images were captured using an OLYMPUS EP 50 camera.^29^

### 2.13 In silico study

#### 2.13.1 Pharmacokinetic and toxicological studies

The pharmacokinetic characteristics of the sort‐out compounds were evaluated using the Swiss ADME (http://www.swissadme.ch/). The primary drug‐likeness parameters studied included molecular weight (MW), hydrogen bond acceptors (HBA), hydrogen bond donors (HBD), violations, lipophilicity (log P), using Lipinski's rules, total polar surface area (TPSA), and number of rotatable bonds (nRB), based on Veber's rules. Concerning the toxicity discovery of the novel medicine, toxicological properties were investigated using the admetSAR program (http://lmmd.ecust.edu.cn/admetsar2/). The following were predicted: Ames toxicity, carcinogenicity, acute oral toxicity, bioavailability, human intestinal absorption, and blood–brain barrier (BBB).^30^

#### 2.13.2 Prediction of Activity Spectra for Substances

Using the *Prediction of Activity Spectra for Substances* (PASS) program, the structures of 10 ME‐CEF compounds were examined for hepatoprotective and antioxidant study. Based on a structure–activity relationship (SAR), the tools forecast the activity spectrum of a molecule as potential activity (Pa) or probable inactivity (Pi) (https://www.way2drug.com/dr/). Values for Pa and Pi fall between 0.000 and 1.000, with pa > pi explaining experimentally active. Pa >0.7 indicates a high level of therapeutic activity.^31^

#### 2.13.3 Molecular docking

##### Protein preparation

Target proteins 2CKJ (xanthin oxidoreductase), 1ALU (IL‐6), 7JRA (TNF‐α), and 7KK6 (PARP‐1) have crystal structures with selective inhibitors at resolutions of 3.59, 1.9, 2.1, and 2.06 Å that can be used to study plant biological activity (https://www.rcsb.org/) by cleaning, making other required preparations, and utilizing Discovery Studio 2024 and the Swiss‐Pdb Viewer. The protein setup included gasteiger charge, energy minimization, and analysis with AMBER ffl4sB and gasteiger mode.^31^

##### Ligand preparation

The 10 bioactive compounds of ME‐CEF were 3,3′‐thiodipropanol (PubChem CID: 66358), cyclodecanone, oxime (PubChem CID: 76303), cyclohexane‐1,2‐diol, 4‐(bicylo[2.2.1]hept‐2‐yl) (PubChem CID: 535167), diethyl 1‐methyl‐3‐hydroxy‐5‐phenylpyrrole‐2,4‐dicarboxylate (PubChem CID: 582209), 2‐furanmethanol, 5‐ethenyltetrahydro‐alpha,alpha,5‐trimethyl (PubChem CID: l6428573), d‐gala‐l‐ido‐octonic amide (PubChem CID:552061), *N*‐heptanoyl‐l‐homoserine lactone (PubChem CID:443437), octyl‐beta‐d‐glucopyranoside (PubChem CID:62852), ethanamine, 1‐(3, 4‐dimethoxyphenyl)‐*N*‐methyl‐*N*‐[2‐(4‐morpholinyl)ethyl] (PubChem CID:585380), 9‐octadecenamide (PubChem CID:1930) selected for molecular docking study. The study compared the docking of ME‐CEF phytocompounds with the standard compound silymarin to assess antioxidant and hepatoprotective activities. Ligands were optimized using the PyRx for target suitability.^32^

##### Molecular docking analysis

Protein and ligand structures were acquired and converted to PDBQT format in this investigation using PyRx Auto Dock Vina. The optimal docking point was determined using a grid box with the following dimensions: *X*: 67.7066 Å, *Y*: 71.9639 Å, and *Z*: 61.6788 Å., BIOVIA Discovery Studio Visualizer 2024 has been used for both two‐dimensional (2D) and three‐dimensional (3D) representations.^33^

### 2.14 Statistical analysis

GraphPad Prism 10.0 was used to examine the statistical data, which were then displayed as mean ± standard error of the mean (SEM). Dunnett's test was performed after a one‐way analysis of variance (ANOVA) to ascertain the statistical significance. When *p < 0.05, **p < 0.01, and ***p < 0.001, values were compared with the control group with statistical significance.

## 3 RESULTS

### 3.1 Yield percentage

After extraction, the percentage yields of ME‐CEF were determined. The result showed that the extract yield was 42.31%.

### 3.2 Acute oral toxicity

This information resulted in the establishment of the 200 and 400 mg/kg BW optimal dosages for the in vivo hepatoprotective study. Throughout the course of this experiment, no negative effects of the extract were noted.

### 3.3 Qualitative analysis

The qualitative analysis of ME‐CEF identified the presence of various secondary metabolites, including alkaloids, carbohydrates, flavonoids, phenols, saponin, tannon, and glycosides, in ME‐CEF (Table 1).

**TABLE 1:** Qualitative phytochemical analysis of ME‐CEF.

Phytochemical constituents	Specific test	Inference
Alkaloids	Mayer's test	++
	Hager test	+++
	Wagner's test	++
Carbohydrates	Molisch's test	+++
	Benedict's test	++
	Fehling's test	++
Flavonoids	Alkaline reagent test	++
Phenols	Ferric chloride test	++
Saponins	Foam test	++
Tannins	Gelatin test	+
Glycoside	Liebermann's test	++

*Note*: Symbol (+++) indicates presence in high concentration; symbol (++) indicates presence in moderate concentration; and symbol (+) indicates presence in trace concentration.

Abbreviation: ME‐CEF, methanolic extract of the *C. esculenta* flower.

### 3.4 Quantitative analysis

The ME‐CEF had acceptable levels of TPC, TFC, and TTC according to the data. The outcome is presented in Table 2.

**TABLE 2:** Quantitative analysis of ME‐CEF.

Total phenolic content	Total flavonoid content	Total tannin content
(mg/g GAE)	(mg/g)	(mg/g)
46.37 ± 2.03	28.26 ± 1.6	12.14 ± 0.8

*Note*: Each value is represented by the mean ± standard error of the mean (SEM) (*n* = 5).

Abbreviation: GAE, gallic acid equivalents; ME‐CEF, methanolic extract of the *C. esculenta* flower.

### 3.5 GC–MS/MS analysis

**FIGURE 1:**
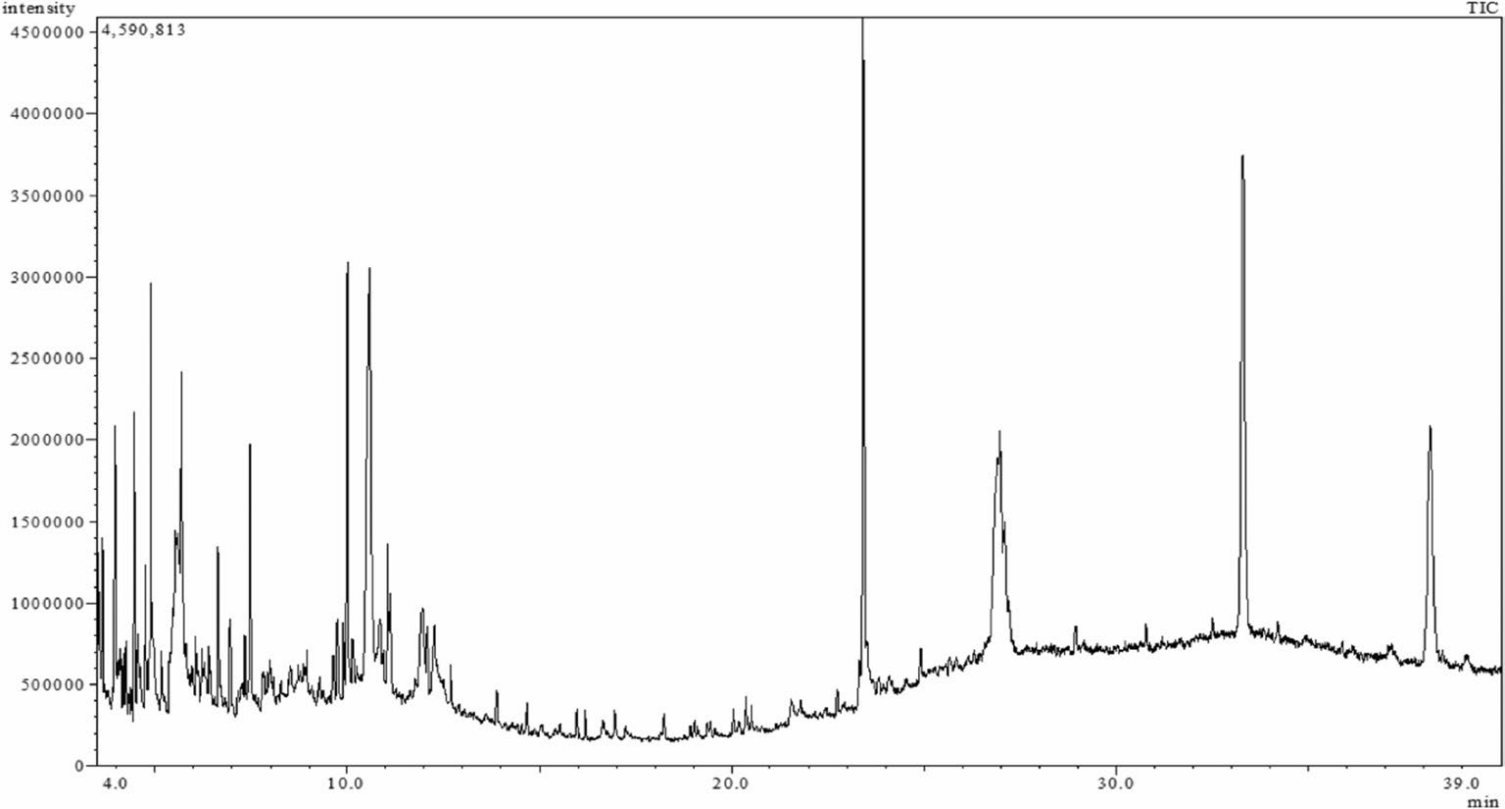
Chromatogram of methanolic extract of the *C. esculenta* flower (ME‐CEF)‐identified compounds.

About 20 chemicals were found in ME‐CEF after GC–MS/MS analysis. The peak areas ranged from 8.65 to 0.2, as shown in Table 3, and the chromatogram is displayed in Figure 1. The major compounds were 2‐(isobutoxymethyl)oxirane (8.65%), 9‐octadecenamide (6.49%), 6‐oxa‐bicyclo[3.1.0]hexan‐3‐one (4.3%), propane, 1,1‐diethoxy‐2‐methyl (2.19%), 5‐methoxy‐3‐phenyl‐1‐pentanol (2.11%), 2‐furanmethanol, 5‐ethenyltetrahydro (2.779%), cyclodecanone, oxime (0.59%), ethanamine, 1‐(3, 4‐dimethoxyphenyl)‐*N*‐methyl‐*N*‐[2‐(4‐morpholyl)ethyl] (0.8%).

**TABLE 3:** Major chemical compounds identified from ME‐CEF.

Sl. no.	Compounds	Formula	MW g/mol	RT	% Area	Structure
01	3‐Allyloxy‐1,2 propanediol	C_6_H_12_O_3_	132.16	3.536	0.41	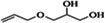
02	Ethanamine, 1‐(3, 4‐dimethoxyphenyl)‐*N*‐methyl‐*N*‐[2‐(4‐morpholyl)ethyl]	C_17_H_28_N_2_O_3_	308.4	4.25	0.84	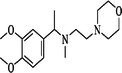
03	6‐Oxa‐bicyclo[3.1.0]hexan‐3‐one	C_5_H_6_O_2_	98.10	4.898	4.3	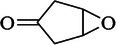
04	Propane, 1,1‐diethoxy‐2‐methyl	C_8_H_18_O_2_	146.23	5.533	2.19	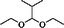
05	2‐Propen‐1‐ol	C_3_H_6_O	58.08	5.682	4.07	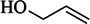
06	Piperidine, 1‐nitroso	C_5_H_10_N_2_O	114.15	6.224	0.71	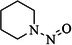
07	d‐Gala‐l‐ido‐octonic amide	C_8_H_17_NO_8_	255.22	7.153	0.24	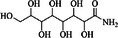
08	5‐Methoxy‐3‐phenyl‐1‐pentanol	C_15_H_26_O_2_Si	194.27	7.485	2.11	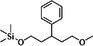
09	Diethyl 1‐methyl‐3‐hydroxy‐5‐phenylpyrrole‐2,4‐dicarboxylate	C_17_H_19_NO_5_	317.34	7.786	0.94	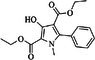
10	3,3’‐Thiodipropanol	C_6_H_14_O_2_S	150.24	8.947	0.45	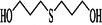
11	Phenol, 2,6‐dimethoxy	C_8_H_10_O_3_	154.16	9.629	0.82	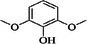
12	2‐Furanmethanol, 5‐ethenyltetrahydro‐alpha,alpha,5‐trimethyl	C_10_H_18_O_2_	170.25	10.004	2.79	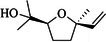
13	2‐(Isobutoxymethyl)oxirane	C_7_H_14_O_2_	130.18	10.579	8.65	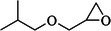
14	3,3‐Dimethylpentanoic acid	C_7_H_14_O_2_	130.18	11.051	0.85	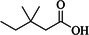
15	Octyl‐beta‐D‐glucopyranoside	C_14_H_28_O_6_	292.37	11.76	0.33	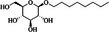
16	3‐Deoxy‐d‐mannoic lactone	C_6_H_10_O_5_	162.14	11.962	2.15	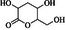
17	Cyclohexane‐1,2‐diol, 4‐(bicylo[2.2.1]hept‐2‐yl)	C_13_H_22_O_2_	210.31	23.325	0.57	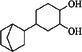
18	9‐Octadecenamide	C_18_H_35_NO	281.5	23.42	6.49	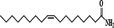
19	Cyclohexanecarboxamide	C_7_H_13_NO	127.18	23.52	0.97	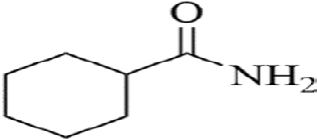
20	Cyclodecanone, oxime	C_10_H_19_NO	169.26	26.265	0.59	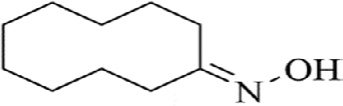

Abbreviations: ME‐CEF, methanolic extract of the *C. esculenta* flower; RT = Retention Time.

### 3.6 Antioxidant activity

#### 3.6.1 ABTS scavenging assay

**FIGURE 2:**
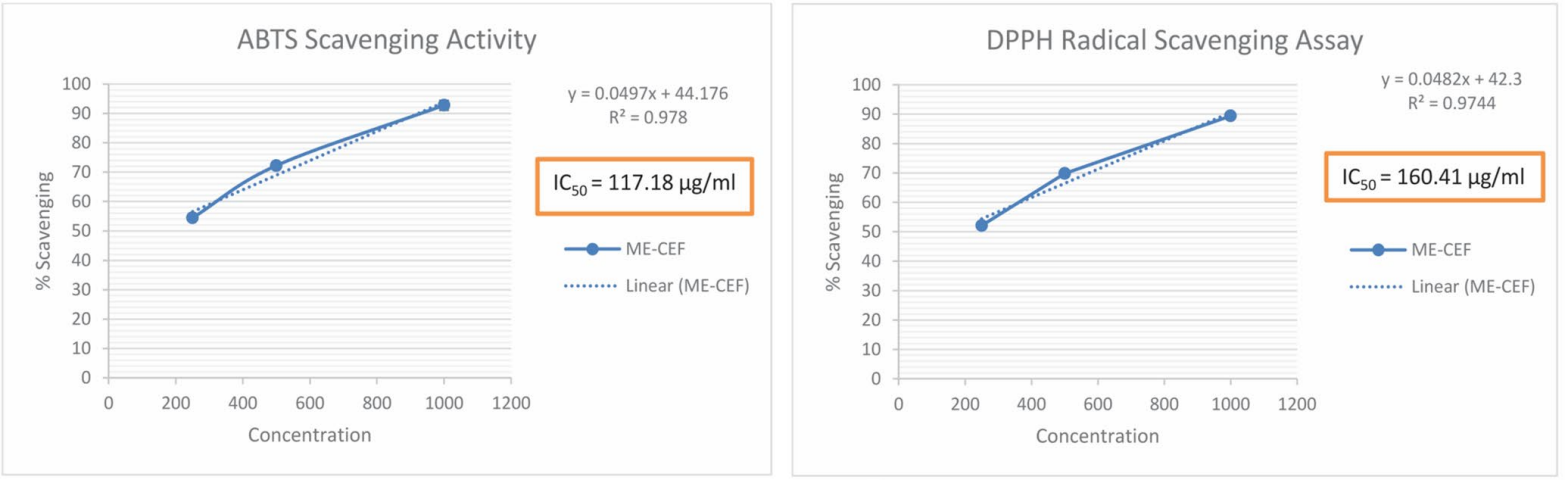
2,2‐Diphenyl‐1‐picrylhydrazyl (DPPH) and 2,2′‐azino‐bis‐(3‐ethylbenzothiazoline‐6‐sulfonic) (ABTS) radical scavenging of methanolic extract of the *C. esculenta* flower (CSF‐ME). The results are expressed as a percentage of reducing activity equivalent to ascorbic acid. Values are expressed as mean ± standard error of the mean (SEM), with *n* = 3.

The results are shown as a concentration curve in Figure 2, with the maximum inhibition percentage of 92.79% ± 1.6% shown for the ME‐CEF 1000 μg/mL concentration. Ascorbic acid, on the contrary, showed greater inhibition at 1000 μg/mL (96.28% ± 0.44%). ME‐CEF has an IC_50_ value of 117.18 μg/mL. Comparing this extract concentration to the standard medication ascorbic acid (18.12 μg/mL), it appears to have a moderate effect on DPPH.

#### 3.6.2 DPPH scavenging activity

A concentration curve illustrating the results is shown in Figure 2, where the concentration of ME‐CEF 1000 μg/mL shows the maximum inhibition percentage of 89.41% ± 1.39%. Ascorbic acid, on the contrary, showed greater inhibition at 1000 μg/mL (96.28% ± 0.44%). ME‐CEF has an IC_50_ value of 160.41 μg/mL. Comparing this extract concentration to the recommended dosage of ascorbic acid (10.12 μg/mL), it appears to have a moderate effect on DPPH.

### 3.7 Hepatoprotective study

#### 3.7.1 Body and organ weight

Table 4 outlines notable BW changes in CCl_4_ and CCl_4_ + 200 mg/kg groups. The CCl_4_ group had the only reduction in final BW, arriving at 32.47 ± 0.68. The group also had the most significant relative liver weight of 7.79 ± 0.56 across all the groups, with their liver weight measuring 2.60 ± 0.86. Among all groups, except the CCl_4_ group, the high‐dose group (CCl_4_ + 400 mg/kg) had the most persistent relative liver weight changes, therefore reducing a significant BW change.

**TABLE 4:** Changes in body and liver weight (g) in various treated groups.

Groups	Initial body weight (g)	Final body weight (g)	% Body weight change	Liver weight (g)	Relative body weight
Control	36.18 ± 1.07	38.15 ± 0.75^ns^	5.49 ± 2.38ꜛ	1.54 ± 0.11	4.02 ± 0.22
CCl_4_	37.19 ± 0.68	32.47 ± 0.68*	−3.44 ± 3.25ꜜ	2.60 ± 0.86^ns^	7.79 ± 0.56
CCl_4_ + silymarin	37.32 ± 0.75	36.46 ± 1.91^ns^	3.11 ± 2.74ꜛ	1.50 ± 0.65^ns^	4.16 ± 0.42
CCl_4_ + ME‐CEF—200	37.74 ± 1.38	39.35 ± 0.97*	10.25 ± 3.90ꜛ	1.99 ± 0.34^ns^	5.02 ± 0.55
CCl_4_+ ME‐CEF—400	36.35 ± 1.51	37.29 ± 1.76^ns^	5.42 ± 4.62ꜛ	1.55 ± 0.54^ns^	4.15 ± 0.42

*Note*: All data are presented as mean ± standard error of the mean (SEM) (*n* = 5). Body weight changes were assessed by comparing final and initial weights within the same group. Liver weight treated and control groups were compared for the difference. ꜛrepresents an increase, ꜜrepresents a decrease.

Abbreviation: ns, nonsignificant.

* *p* < 0.05.

#### 3.7.2 Lipid profile

Table 5 shows a substantial increase in CHO, TG, and LDL levels by 233.24%, 168.50%, and 564.9%, respectively, with a 40.7% decrease in HDL levels compared to the control group due to CCl_4_ induction. However, the standard and treatment groups (200 and 400 mg/kg BW) successfully restored all the lipid profile indicators compared to the CCl_4_ group. Specifically, the treatment group exhibited a reduction of 39.14% in CHO, 47.95% in TG, and 65.74% in LDL, whereas the CCl_4_ + 200 mg/kg group showed a decrease of 3.19%, 39.10%, and 62.15%, and the CCl_4_ + 400 mg/kg group exhibited reduction of 24.85%, 58.75%, and 78.09%. Moreover, HDL levels were increased by 47.95% for the treatment group, 39.10% for CCl_4_ + 200 mg/kg, and 50.52% for the CCl_4_ + 400 mg/kg BW group.

**TABLE 5:** Effect of ME‐CEF on lipidic profile and biochemical markers.

Activity		Control	Normal control + CCl_4_	Silymarin + CCl_4_	CCl_4_ + ME‐CEF—200 mg/kg	CCl_4_ + ME‐CEF—400 mg/kg
Liver function test	ALT (U/L)	80.66 ± 4.21	332.66 ± 12.21***	108.23 ± 6.47***	195.16 ± 8.27***	152.90 ± 7.32***
	AST (U/L)	109.86 ± 3.89	278.83 ± 8.77***	135.8 ± 3.29***	216.23 ± 10.32***	161.8 ± 6.35***
	ALP (U/L)	45.8 ± 3.33	241.83 ± 6.55***	114.13 ± 4.42***	203.13 ± 7.65**	179.93 ± 6.88***
	BILI (mg/dL)	0.48 ± 0.06	2.31 ± 0.08***	0.77 ± 0.05***	1.95 ± 0.08***	1.73 ± 0.05***
RFT	CRE (mg/dL)	0.403 ± 0.012	3.16 ± 0.06***	1.75 ± 0.08***	2.87 ± 0.08**	1.23 ± 0.015***
Lipid profile (mg/dL)	CHO	57.69 ± 0.84	192.25 ± 5.82***	78.65 ± 2.86***	125.12 ± 3.24***	97.12 ± 2.04***
	TG	45.50 ± 1.90	122.17 ± 7.46***	63.58 ± 3.16***	74.39 ± 3.07***	50.39 ± 4.91***
	HDL	30.78 ± 1.19	18.25 ± 4.35*	24.66 ± 1.76*	19.47 ± 3.52^ns^	27.47 ± 2.80**
	LDL	8.17 ± 0.87	54.33 ± 2.08***	18.61 ± 0.88***	20.56 ± 1.35***	11.9 ± 1.26***

*Note*: All values are represented by mean ± standard error of the mean (SEM). Control versus CCl_4_, CCl_4_ versus CCl_4_ + silymarin, CCl_4_ versus CCl_4_ + ME‐CEF—200, CCl_4_ versus CCl_4_ + ME‐CEF—400.

Abbreviations: ALP, alkaline phosphatase; ALT, amino transferase; AST, aspartate amino transferase; BILI, bilirubin; CHO, cholesterol; CRE, creatinine; HDL, high‐density lipoprotein; LDL, low‐density lipoprotein; ns, nonsignificant, RFT, renal function test; TG, triglyceride.

* *p* < 0.05;

** *p* < 0.01;

*** *p* < 0.001.

#### 3.7.3 Biochemical marker

**FIGURE 3:**
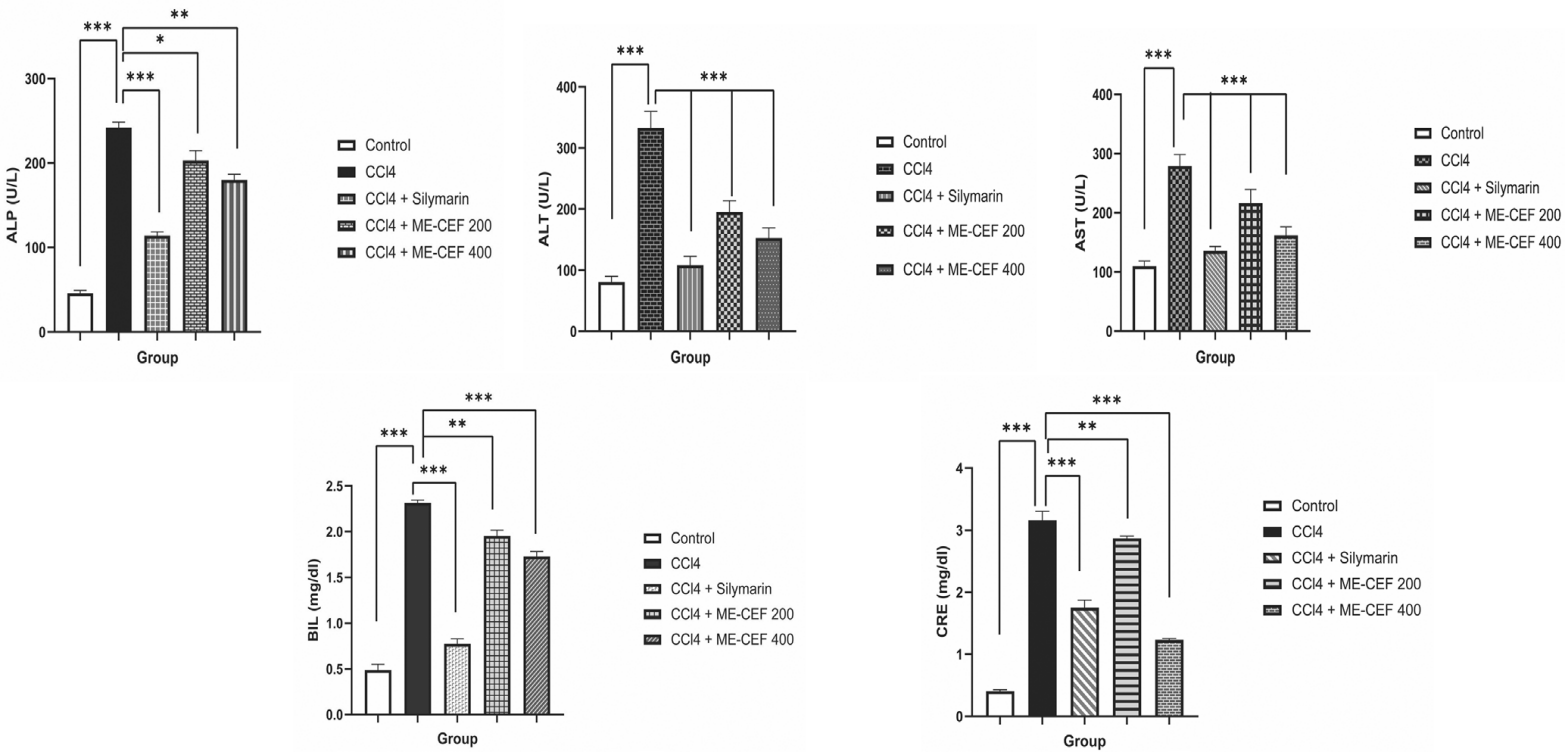
Biochemical estimation of alkaline phosphatase (ALP), amino transferase (ALT), aspartate amino transferase (AST), bilirubin, and creatinine. All values are represented by mean ± standard error of the mean (SEM). ns, nonsignificant, *p < 0.05, **p < 0.01, ***p < 0.001. Control versus CCl4, CCl4 versus CCl4 + silymarin, CCl4 versus CCl4 + CSF‐ME—200, CCl4 versus CCl4 + CSF‐ME—400.

The five serum biochemical markers were observed in the CCl_4_‐treated liver‐injured mice. The CCl_4_‐treated mice had a significant enhancement (*p* < 0.001) in ALT, AST, ALP, bilirubin, and creatinine by 312.42%, 153.80%, 428.01%, 381.25%, and 684.11% compared to the control group. However, compared to the CCl_4_ group, the standard and treated groups (200 and 400 mg/kg BW) exhibited decreases in the levels of ALT, AST, ALP, bilirubin, and creatine. The reductions were as follows: standard group: by 67.46%, 51.29%, 52.80%, 66.6%, and 44.62%, respectively; CCl_4_ + ME‐CEF 200 mg/kg group: 41.33%, 22.45%, 16.01%, 15.58%, and 9.17%, respectively; CCl_4_+ ME‐CEF 400 mg/kg group: 54.03%, 41.97%, 25.59%, 25.10%, and 39.92%, respectively (Figure 3).

#### 3.7.4 Catalase activity

**FIGURE 4:**
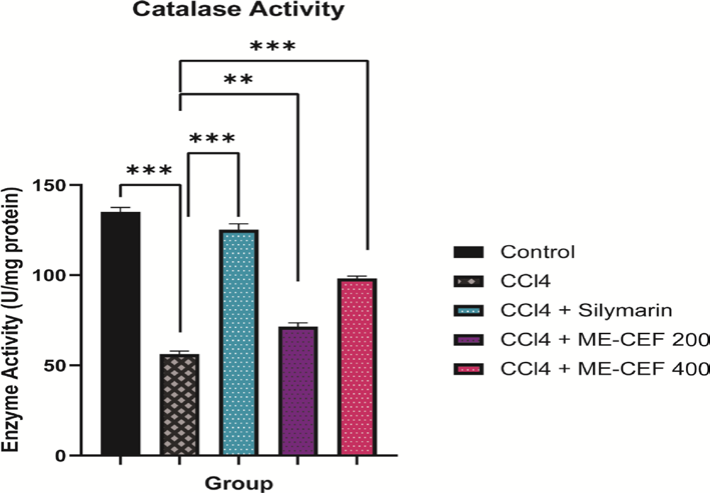
Catalase activity of methanolic extract of the *C. esculenta* flower (ME‐CEF). All values are represented by mean ± standard error of the mean (SEM). **p < 0.01, ***p < 0.001. Control versus CCl_4_, CCl_4_ versus CCl_4_ + silymarin, CCl_4_ versus CCl_4_ + ME‐CEF—200, CCl_4_ versus CCl_4_ + ME‐CEF—400. ME‐CEF, methanolic extract of the *C. esculenta* flower.

The activity of the hepatic antioxidant enzyme CAT was found to be drastically decreased (*p* < 0.001) in the CCl4 group compared to the standard group. This resulted in a 41.58% lowering of antioxidant enzyme activity. This protective effect was more marked in mice with 100 mg/kg of silymarin (92.63%). The decreased activity of the enzymes of intoxicated mice was augmented more effectively with CCl4 + ME‐CEF 400 mg/kg, showing 72.56% (Figure 4).

#### 3.7.5 Histopathological study

**FIGURE 5:**
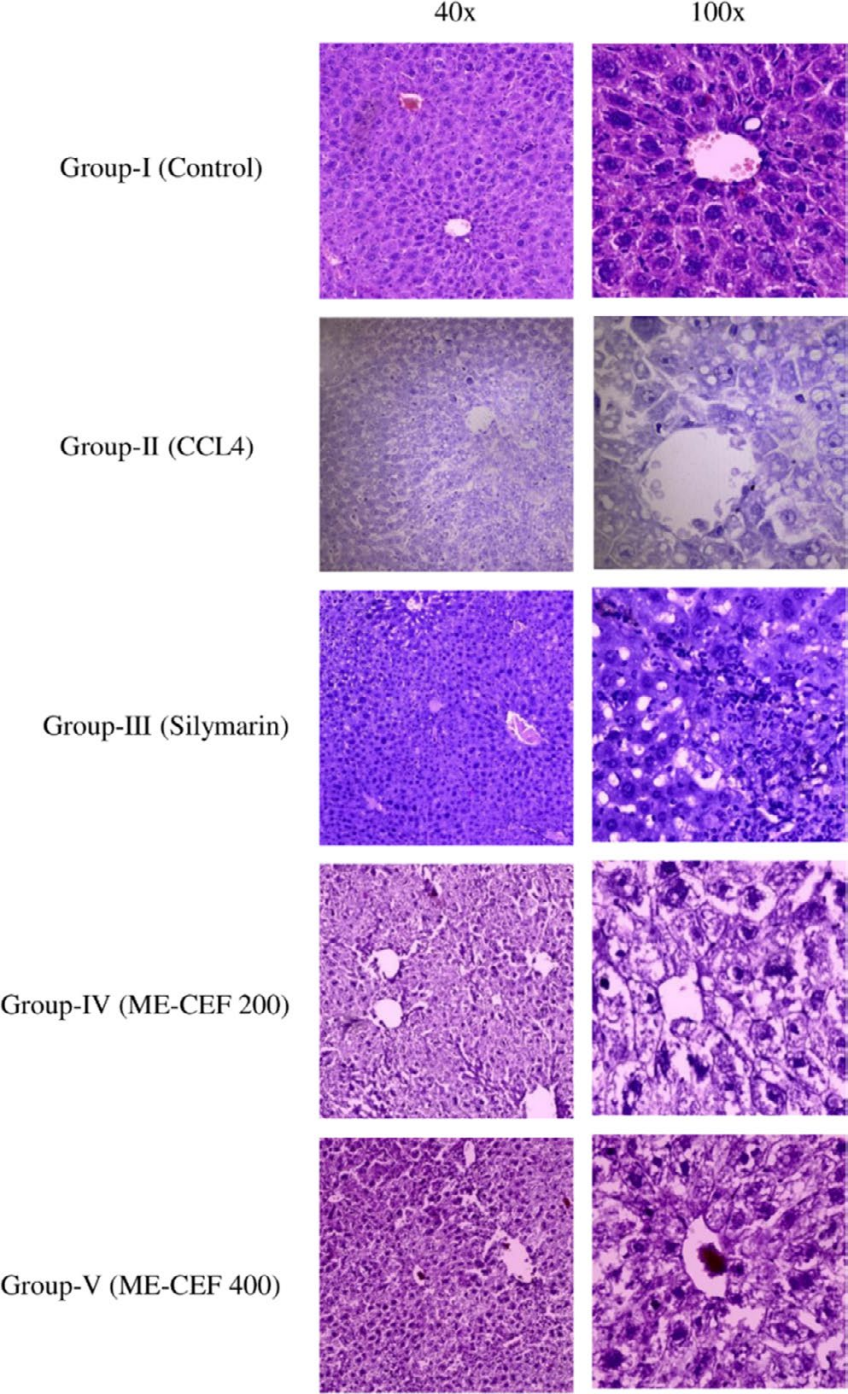
Photomicrographs (40× and 100×) of the histopathological examination of the liver samples of different groups (control, CCl_4_, CCl_4_ + silymarin, CCl_4_ + ME‐CEF—200, CCl_4_ + ME‐CEF—400). ME‐CEF, methanolic extract of the *C. esculenta* flower.

Figure 5 depicts the results of the histological examination using the control and the treated groups. The control group's liver possessed intact architectural orientation, uniform hexagonal cells with rounded nuclei, granular nuclear chromatin and abundant cytoplasm, and few congested blood vessels. CCL_4_ group's liver possessed architecture loss in multiple floral areas, mild inflammatory cells, infiltrate in perivascular area, and vascular change in few hepatocytes with enlarged nuclear size. Silymarin group's liver samples showed mostly normal architecture, with floral area showing the loss of architectural orientation, nuclear hyperchromasia with increased vascular proliferation, and more congested blood vessels. Inflammation was present, along with signs of regeneration such as nuclear division. Hepatocytes near the vascular area appeared normal and regenerative, whereas those comparatively away from the vascular area showed toxic changes. CCl4 + ME‐CEF 200 group showed the loss of orientation of hepatocytes with severe vascular change, mild infiltration of chronic inflammatory cells (lymphocyte) in the perivascular area and intercellular space, more cytoplasmic (vascular change) than nuclear changes, and more vascular changes. CCl4 + ME‐CEF 400 group showed moderate architectural orientation; vascular changes in the cells were more prominent, with the loss of nucleus in many hepatocytes and more congestion with increased infiltration of chronic inflammatory cells.

### 3.8 In silico study

#### 3.8.1 ADME/T study

Every phytocompound was extracted for drug‐likeliness and ADME/T assessment. Of the 20 compounds, 10 are in compliance with Veber's and Lipinski's rule of five, whereas 10 violate both sets of regulations.

Ten of the phytocompounds met the requirements after being put through additional toxicity testing. After screening for Ames toxicity, acute oral toxicity, carcinogenesis, and BBB permeability, the compounds demonstrated optimal solubility and a high rate of gastrointestinal absorption. Every other compound adhered to Lipinski's five‐point criterion. AdmetSAR was used to evaluate the toxicity of the data screening, with silymarin serving as the benchmark (Tables 6 and 7).

**TABLE 6:** ADME/T property prediction of the bioactive compounds of ME‐CEF.

ADME							
Compound name	Lipinski's rules				Lipinski's violations ≤ 1	Veber's rules	
	MW (g/mol) < 500	HBA < 10	HBD < 5	Log *p* ≤ 5		nRB ≤ 10	TPSA ≤ 140
3,3′‐Thiodipropanol	257.28	3	1	2.08	0	2	59.30 Å^2^
Cyclodecanone, oxime	169.26	2	1	2.31	0	0	32.59 Å^2^
Cyclohexane‐1,2‐diol, 4‐(bicylo[2.2.1]hept‐2‐yl)‐	210.31	2	2	2.32	0	1	40.46 Å^2^
Diethyl,1‐methyl‐3‐hydroxy‐5‐phenylpyrrole‐2,4‐dicarboxylate	317.34	5	1	3.29	0	7	77.76 Å^2^
2‐Furanmethanol, 5‐ethenyltetrahydro‐alpha,alpha,5‐trimethyl	170.25	2	1	2.45	0	2	29.46 Å^2^
d‐Gala‐l‐ido‐octonic amide	255.22	8	8	−0.09	1	7	184.70 Å^2^
*N*‐Heptanoyl‐l‐homoserine lactone	213.27	3	1	2.15	0	7	55.40 Å^2^
Octyl‐.beta.‐d‐glucopyranoside	292.37	6	4	1.42	0	9	99.38 Å^2^
Ethanamine, 1‐(3, 4‐dimethoxyphenyl)‐*N*‐methyl‐*N*‐[2‐(4‐morpholyl)ethyl]	308.42	5	0	3.55	0	7	34.17 Å^2^
9‐Octadecenamide	281.48	1	1	4.22	1	15	43.09 Å^2^

Abbreviations: HBA, hydrogen bond acceptor; HBD, hydrogen bond donor; log P, lipophilicity; ME‐CEF, methanolic extract of the *C. esculenta* flower; MW, molecular weight; nRB, number of rotatable bonds; TPSA, topological polar surface area.

**TABLE 7:** Toxicological study of selected bioactive compounds from ME‐CEF.

Toxicity study						
Compound name	Ames toxicity	Carcinogens	Acute oral toxicity	Human intestinal absorption	Human oral bioavailability	Blood–brain barrier
3,3′‐Thiodipropanol	NAT	NC	III	0.9849	0.6000	0.7250
Cyclodecanone, oxime	NAT	NC	III	0.9327	0.7000	0.9250
Cyclohexane‐1,2‐diol, 4‐(bicylo[2.2.1]hept‐2‐yl)	NAT	NC	III	0.9827	0.6143	0.5750
Diethyl,1‐methyl‐3‐hydroxy‐5‐phenylpyrrole‐2,4‐dicarboxylate	NAT	NC	III	0.9136	0.6857	0.5750
2‐Furanmethanol, 5‐ethenyltetrahydro‐alpha,alpha,5‐trimethyl	NAT	NC	III	0.9774	0.5857	0.8750
d‐Gala‐l‐ido‐octonic amide	NAT	NC	III	0.6012	0.5714	0.6000
*N*‐Heptanoyl‐l‐homoserine lactone	NAT	NC	III	0.9846	0.5714	0.7500
Octyl‐beta‐d‐glucopyranoside	NAT	NC	III	0.7714	0.7429	0.5500
Ethanamine, 1‐(3, 4‐dimethoxyphenyl)‐*N*‐methyl‐*N*‐[2‐(4‐morpholyl)ethyl]	NAT	NC	III	0.9195	0.8571	0.9250
9‐Octadecenamide	NAT	NC	III	0.9964	0.5429	0.9750

Abbreviations: ME‐CEF, methanolic extract of the *C. esculenta* flower; NAT, not Ames Toxicity; NC, noncarcinogenic.

#### 3.8.2 Forecast for passing

The PASS online program was used to analyze 10 distinct components to evaluate the plant's antioxidant and hepatoprotective properties. The potent compound has shown greater hepatoprotective and antioxidant activity of Pa values compared to Pi values. Although eight compounds (3,3′‐thiodipropanol, d‐gala‐l‐ido‐octonic amide, diethyl 1‐methyl‐3‐hydroxy‐5‐phenylpyrrole‐2,4‐dicarboxylate, *N*‐heptanoyl‐l‐homoserinelactone, octyl‐beta‐d‐glucopyranoside, 2‐furanmethanol, 5‐ethenyltetrahydro‐alpha,alpha,5‐trimethyl, cyclodecanone, oxime, ethanamine, 1‐(3, 4‐dimethoxyphenyl)‐*N*‐methyl‐*N*‐[2‐(4‐morpholyl)ethyl]) showed both antioxidant and hepatoprotective activities, and cyclohexane‐1,2‐diol, 4‐(bicylo[2.2.1]hept‐2‐yl) showed antioxidant activity and 9‐octadecenamide showed hepatoprotective activity among the compounds, octyl‐beta‐D‐glucopyranoside showed the highest Pa value in both antioxidant and hepatoprotective activities (0.600 and 0.831). The outcome is presented in Table 8.

**TABLE 8:** PASS prediction of selected ME‐CEF compounds for antioxidant and hepatoprotective effects.

Compounds	Antioxidant		Hepatoprotective	
	Pa	Pi	Pa	Pi
3,3′‐Thiodipropanol	0.181	0.097	0.194	0.026
d‐Gala‐l‐ido‐octonic amide	0.132	0.124	0.312	0.131
Diethyl 1‐methyl‐3‐hydroxy‐5‐phenylpyrrole‐2,4‐dicarboxylate	0.450	0.007	0.654	0.042
*N*‐Heptanoyl‐l‐homoserine lactone	0.510	0.004	0.243	0.092
Octyl‐beta‐d‐glucopyranoside	0.600	0.005	0.831	0.004
2‐Furanmethanol, 5‐ethenyltetrahydro‐alpha,alpha,5‐trimethyl	0.623	0.004	0.304	0.058
Cyclohexane‐1,2‐diol, 4‐(bicylo[2.2.1]hept‐2‐yl	0.406	0.020	–	–
Cyclodecanone, oxime	0.440	0.015	0.171	0.167
Ethanamine, 1‐(3, 4‐dimethoxyphenyl)‐*N*‐methyl‐*N*‐[2‐(4‐morpholyl)ethyl	0.203	0.073	0.054	0.164
9‐Octadecenamide	–	–	0.450	0.025

*Note*: “Pa” indicates “probability to be active,” and “Pi” indicates “probability to be inactive.”

Abbreviation: ME‐CEF, methanolic extract of the *C. esculenta* flower.

#### 3.8.3 Molecular docking

**FIGURE 6:**
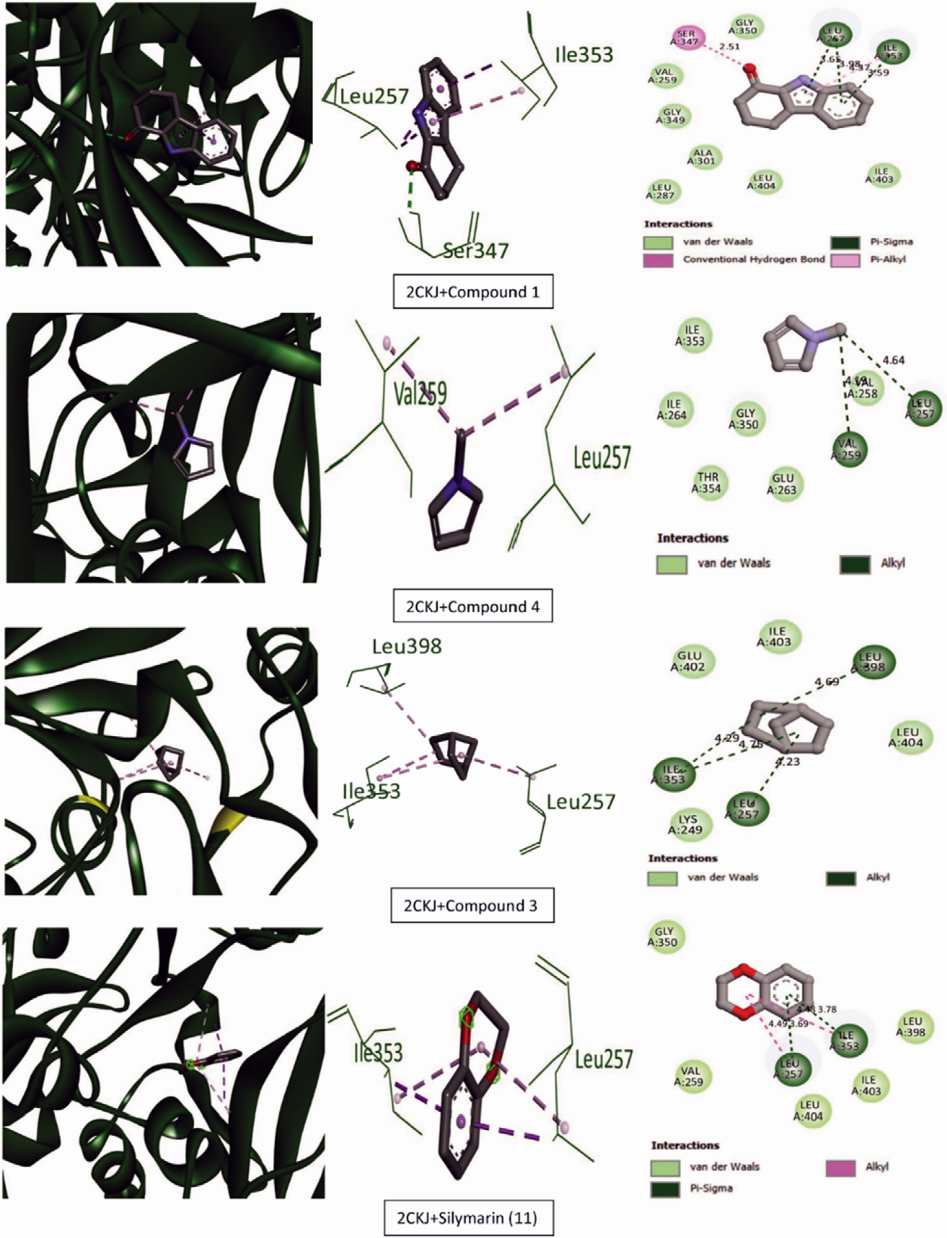
Graphical representation of the molecular interactions of the most prominent bioactive with the 2CKJ (xanthin oxidoreductase) enzyme with three‐dimensional (3D) visualization.

**FIGURE 7:**
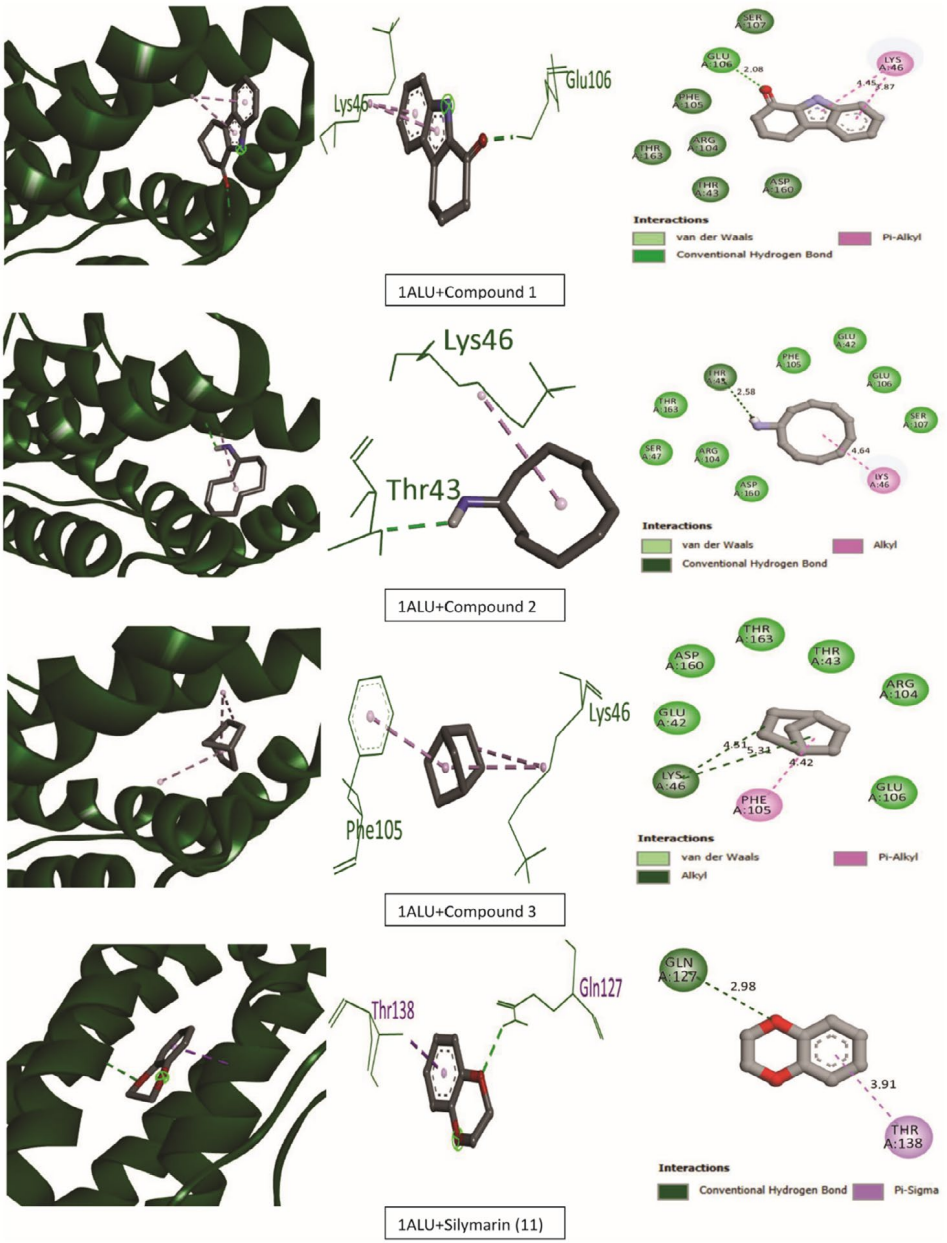
Graphical representation of the molecular interactions of the most prominent bioactive with the 1ALU (interleukin‐6) enzyme with three‐dimensional (3D) visualization.

**FIGURE 8:**
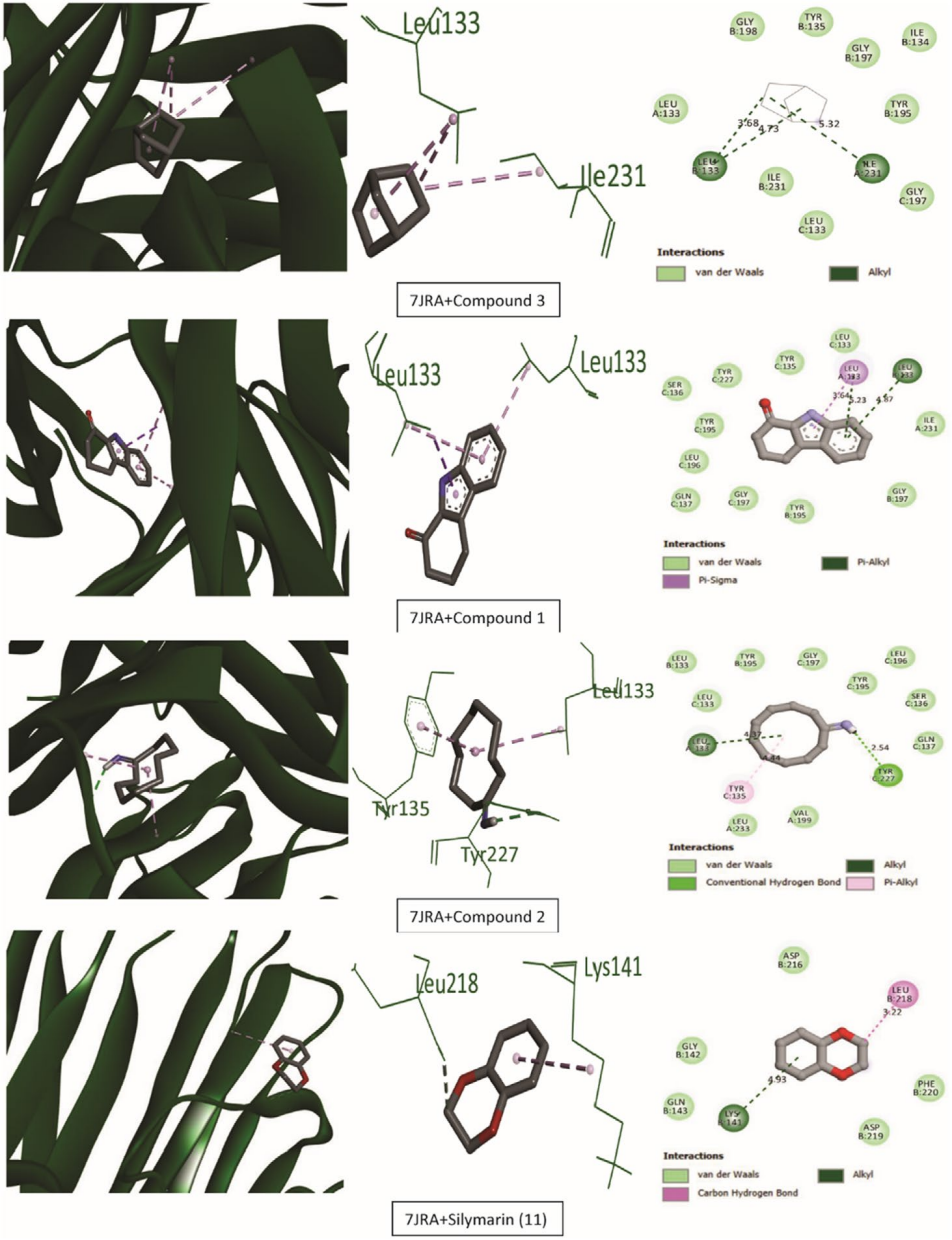
Graphical representation of the molecular interactions of the most prominent bioactive with the 7JRA (tumor necrosis factor α [TNF‐α]) enzyme with three‐dimensional (3D) visualization.

**FIGURE 9:**
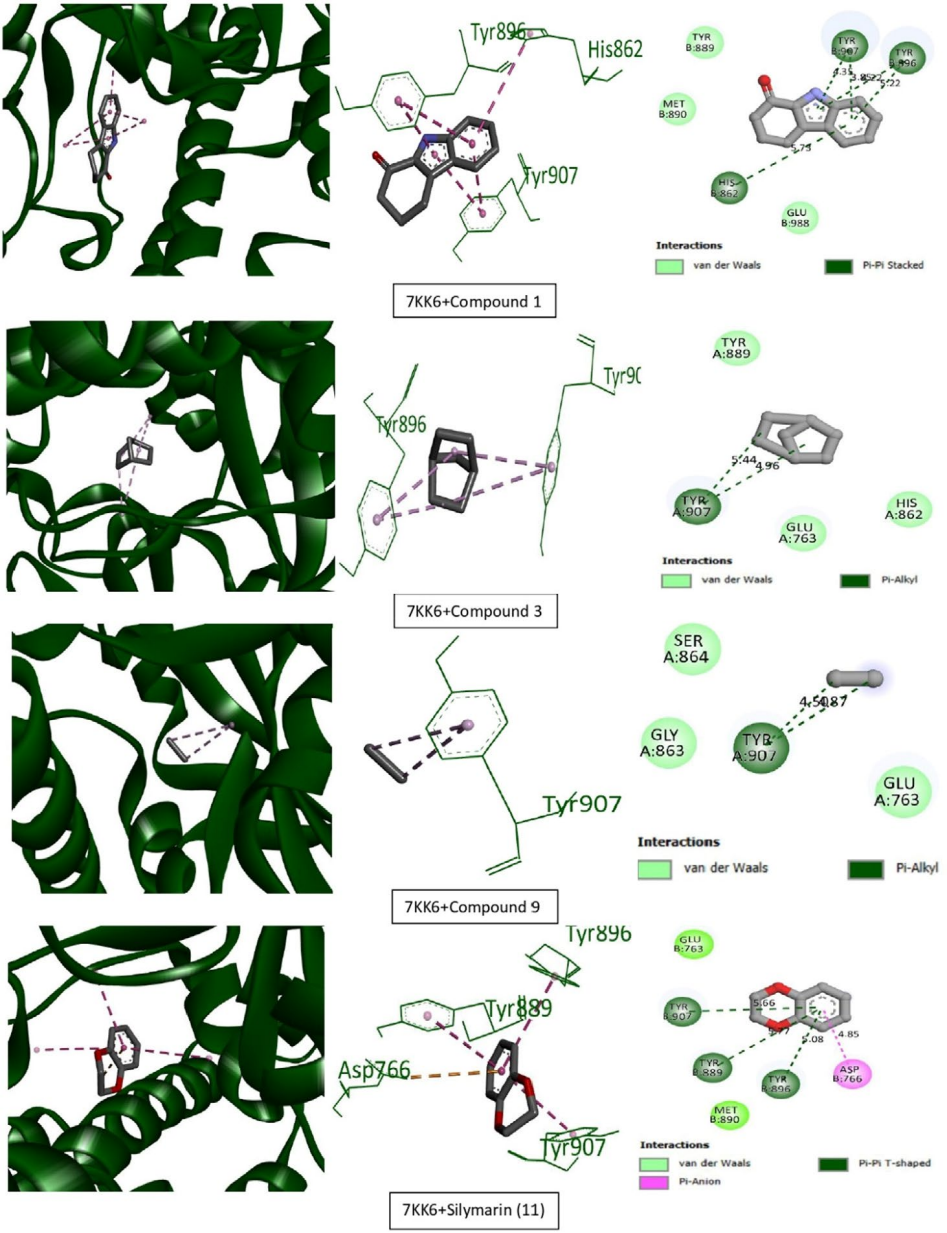
Graphical representation of the molecular interactions of the most prominent bioactive with the 7KK6 (PRAP‐1) enzyme with three‐dimensional (3D) visualization.

Tables 9 and 10 provide an overview of the ME‐CEF molecular docking experiment. The active compounds' docking assessment was applied to the active sites 1ALU (IL‐6), 7JRA (TNF‐α), 2CKJ (xanthin oxidoreductase) for antioxidant activity, and 7KK6 (PARP‐1) for hepatoprotective activity. Grid docking analysis was conducted using PyRx AutoDock Vina to examine the interactions of all 10 chemicals with standards. In comparison to the standard drug silymarin (which has a binding affinity of −10.2 against 2CKJ, −7.8 against 1ALU, −8 against 7JRA, and −10.4 against 7KK6), our study has demonstrated that 3,3′‐thiodipropanol has the highest affinity against antioxidant and hepatoprotective activity (−8.1 against 2CKJ, −6.4 against 1ALU, −8.2 against 7JRA, and −9.1 against 7KK6). Compounds with the highest binding affinity with standard compounds are shown visually in two and three dimensions (Figures 6, 7, 8, 9).

**TABLE 9:** Molecular docking study of ME‐CEF selected compounds.

Sl no.	Compounds	Binding score			
		2CKJ	1ALU	7JRA	7KK6
1	3,3′‐Thiodipropanol	**−8.1**	**−6.4**	**−8.2**	**−9.1**
2	Cyclodecanone, oxime	**−**6.1	**−**5.9	**−**7.9	**−**7
3	Cyclohexane‐1,2‐diol, 4‐(bicylo[2.2.1]hept‐2‐yl)	**−**7.6	**−**5.7	**−**8.5	**−**7.7
4	Diethyl 1‐methyl‐3‐hydroxy‐5‐phenylpyrrole‐2,4‐dicarboxylate	**−**7.8	**−**5.3	**−**5.9	**−**5.7
5	2‐Furanmethanol, 5‐ethenyltetrahydro‐alpha,alpha,5‐trimethyl	**−**6	**−**5.2	**−**6.7	**−**5.4
6	d‐Gala‐l‐ido‐octonic amide	**−**6.2	**−**5.2	**−**6.2	**−**5.7
7	*N*‐Heptanoyl‐l‐homoserine lactone	**−**6.5	**−**5.1	**−**5.9	**−**6.1
8	Octyl‐beta‐d‐glucopyranoside	**−**7	**−**5	**−**5.5	**−**6.9
9	Ethanamine, 1‐(3, 4‐dimethoxyphenyl)‐*N*‐methyl‐*N*‐[2‐(4‐morpholyl)ethyl]	**−**6.6	**−**5.1	**−**6.8	**−**7.2
10	9‐Octadecenamide	**−**6.5	**−**4.4	**−**7.6	**−**6.1
11	Standard silymarin	**−**10.2	**−**7.1	**−**8	**−**10.4

*Note*: 2CKJ (xanthin oxidoreductase) for antioxidant, 1ALU (interleukin‐6), 7JRA (tumor necrosis factor α [TNF‐α]), and 7KK6 (PARP‐1) for hepatoprotective activity compared with standard silymarin enzymes. Bold indicates the highest value.

Abbreviation: ME‐CEF, methanolic extract of the *C. esculenta* flower.

**TABLE 10:** Binding site evaluation of ME‐CEF selected compounds based on molecular docking study.

Sl. no.	Receptor	Compounds	Binding affinity (kcal/mol)	Bond type	Amino acids
1	1ALU	1	**−**6.4	Conventional hydrogen bond	GLU106
				Pi‐alkyl	LYS46, LYS46
		2	**−**5.9	Conventional hydrogen bond	THR43
				Alkyl	LYS46
		3	**−**5.7	Alkyl	LYS46
				Alkyl	LYS46
				Pi‐alkyl	PHE10
		4	**−**5.3	Pi‐anion	ASP26
		Silymarin	**−**7.1	Conventional hydrogen bond	GLN127, HE21
				Pi‐sigma	THR138
2	2CKJ	1	**−**8.1	Conventional hydrogen bond	SER347
				Pi‐sigma	LEU257, LEU257, ILE353
				Pi‐alkyl	ILE353
		4	**−**7.8	Alkyl	LEU257, VAL259
		2	**−**7.6	Conventional hydrogen bond	LEU121
				Alkyl	PRO84
				Pi‐alkyl	TYR121
		8	**−**7	Alkyl	ILE264
		Silymarin	**−**10.2	Pi‐sigma	LEU257, ILE353
				Alkyl	LEU257, ILE353
3	7JRA	3	**−**8.5	Alkyl	LEU133, ILE231, LEU133
		1	**−**8.2	Pi‐sigma	LEU133
				Pi‐alkyl	LEU133, LEU133
		2	**−**7.9	Conventional hydrogen bond	TYR227
				Alkyl	LEU133
				Pi‐alkyl	TYR135
		10	**−**7.6	Conventional hydrogen bond	TYR195
				Pi‐donor hydrogen bond	TYR135
				Alkyl	LEU133, ILE231, LEU133, LEU133, ILE231, LEU133
		Silymarin	**−**8	Carbon hydrogen bond	LEU218
				Alkyl	LYS141
4	7KK6	1	**−**9.1	Pi–Pi stacked	HIS862, TYR896, TYR907, TYR896, TYR907
		3	**−**7.7	Pi‐alkyl	TYR896, TYR896, TYR907, TYR907
		9	**−**7.2	Pi‐alkyl	TYR907, TYR907
		2	**−**7	Pi‐sigma	TYR896
				Pi‐alkyl	TYR907
		Silymarin	**−**10.4	Pi‐anion	ASP766
				Pi–Pi T‐shaped	TYR889, TYR896, TYR907

## 4 DISCUSSION

The liver plays a vital role and regulates various physiological processes (growth, nutrition, and metabolism) and physicochemical functions (oxidation, reduction, and conjugation).^34^ Liver injury can arise from different factors, such as viral infection, drug overdose, alcohol, xenobiotics, and chemicals. Inflammation and oxidative stress can trigger acute liver injury.^35^ Carbon tetrachloride (CCl_4_) is an organic xenobiotic known for inducing free radicals, inflammation, and acute liver injury. Antioxidants are crucial in mitigating CCl_4_‐induced liver damage by counteracting free radicals, lipid peroxidation, and pro‐inflammatory cytokines.^36^ Phenol and flavonoid compounds in plants exhibit strong antioxidant effects by reducing oxidative damage caused by free radicals and ROS during metabolic reactions.^37^ In our initial analysis, we demonstrated the existence of various secondary metabolites of ME‐CEF along with approximate TPC, TFC, and TTC levels at around 46.37 mg/g GAE, 28.26 mg/mL QE, 12.14 mg/g TAE. Using GC–MS/MS analysis, 20 compounds with percentage peak areas ranging from 8.65 to 0.27 were found; however, some chromatogram peaks are still unknown, which may provide information for the characterization of phytochemicals.

Liver injury has diverse etiologies, including viral infections, drug overdoses, alcohol abuse, and toxin exposure. CCl_4_, a chlorinated hydrocarbon, is a potent hepatotoxin. CCl_4_ is metabolized by cytochrome P_450_ enzymes, generating the trichloromethyl radical (CCl_3_•), which reacts with ROS to form the trichloromethyl peroxyl radical (OOCCl_3_•).^38^ These radicals increase oxidative stress, triggering lipid peroxidation and inflammation, ultimately leading to liver damage.^39^, ^40^ These radicals increase oxidative stress, leading to lipid peroxidation and glutathione system disruption, which promote pro‐inflammatory cytokine release, including TNF‐α and IL‐6, resulting in liver damage.^41^ CCl_4_‐induced liver injury serves as a reliable experimental model, closely mimicking human liver disease and aiding therapeutic strategy development.^42^ Antioxidants are vital for reducing cellular damage and apoptosis.^43^ Both the ABTS and DPPH assays are effective for assessing antioxidant capacity due to their ability to measure the scavenging activity of antioxidants against specific free radicals, which is a key indicator of antioxidant potential.^44^ We assessed their efficacy using DPPH and ABTS assays to better understand their ability to counteract oxidative stress in cells. The results showed that ME‐CEF had significant ability to scavenge free radicals compared to ascorbic acid. Conversely, our study's findings revealed that ME‐CEF contains secondary metabolites like tannins, flavonoids, and phenols, many of which are known for their antioxidant and hepatoprotective properties.^45^ Therefore, we aimed to explore the potential antioxidant and hepatoprotective effects of these phenolic compounds.

A combination of serum biochemical markers, oxidative stress parameters, antioxidant enzymes, and immunohistochemical staining of liver tissue is commonly employed to assess the antioxidant and hepatoprotective properties of various compounds in animal models of liver injury.^46^ The administration of ME‐CEF significantly lowered elevated AST, ALT, LDH, ALP, bilirubin, and gamma‐glutamyl transferase (GGT) levels, suggesting its protective role against CCl_4_‐induced liver damage.^47^ This hepatoprotective effect may arise from its ability to preserve liver cell membrane integrity and enhance overall liver function.^48^ Hepatocytes possess antioxidant enzymes like CAT to combat CCl_4_‐induced damage.^49^ However, excessive ROS production can overwhelm these defenses, leading to cellular injury and inflammation via pro‐inflammatory cytokines like TNF‐α, IL‐1, and IL‐6. Pretreatment with ME‐CEF extract mitigated CCl_4_‐induced oxidative stress by enhancing CAT activity and reducing protein oxidation and lipid peroxidation.^50^

The study found that CCl_4_ exposure significantly altered the lipid profile, but pretreatment with ME‐CEF mitigated these changes in mouse plasma. This hypolipidemic effect may result from the inhibition of pancreatic lipase, essential for fat digestion, and hydroxymethylglutaryl‐coenzyme A (HMG‐CoA) reductase, a key enzyme in cholesterol synthesis, leading to reduced cholesterol levels.^51^ Additionally, ME‐CEF increased HDL‐C levels by suppressing the SR‐BI receptor, which is known to stimulate HDL production.^52^ The decrease in LDL levels after ME‐CEF pretreatment may be due to the protection of hepatic LDL‐R genes, enhancing the clearance of LDL from circulation and lowering blood LDL levels.^53^

Histological analysis revealed that pretreatment with ME‐CEF significantly reduced centrilobular necrosis and inflammatory infiltration in animals exposed to CCl_4_ toxicity. These findings indicate that ME‐CEF may help alleviate liver damage caused by CCl_4_, likely due to its bioactive components. Lipinski's rule of five and Veber's rule provide a useful framework for assessing the drug‐likeness and oral bioavailability of potential drug candidates during early drug discovery.^54^ The ADME/T predicts 20 bioactive compounds, of which 10 do not violate Lipinski's and Veber's rule and toxicity. PASS is a computer‐based approach that predicts the biological activity spectrum of a compound based on its structural formula.^55^ After passing the prediction filters, the 10 selected bioactive compounds showed high probability of both antioxidant and hepatoprotective activities.

This study utilized molecular docking simulations to explore the interactions between various phytocompounds in ME‐CEF and the binding sites of four enzymes: xanthine oxidoreductase, PARP‐1, TNF‐α, and IL‐6.^56^ PRAP‐1 is highly expressed in liver epithelial cells but downregulated in hepatocellular carcinoma. It supports hepatoprotection by reducing oxidative stress, promoting liver regeneration, modulating inflammation, and enhancing cellular defense. These functions help maintain liver health, prevent damage, and contribute to overall liver function and resilience against injury.^57^ The analysis aimed to predict binding modes and elucidate their hepatoprotective effects. Ten identified compounds, including 3,3′‐thiodipropanol and cyclodecanone oxime, demonstrated favorable binding to the active sites of the enzymes, with binding energy scores ranging from −4.4 to −9.1 Kcal/Mol, comparable to the reference drug silymarin. Notably, specific residues, such as HIS862, TYR896, and TYR907 in PARP‐1; LEU133, TYR227, and LYS188 in IL‐6; ASN168 for TNF‐α; ASN168, SER347, LEU257, and ILE353 in xanthine oxidoreductase, were crucial for inhibition. These findings suggest that these residues are vital for the inhibition of inflammation‐related proteins. The interactions, particularly hydrogen bonding between these residues and ME‐CEF compounds, highlight the extract's promising anti‐inflammatory and hepatoprotective potential.

Our study is constrained by the same restrictions that apply to all scientific investigations. First, the fact that this study solely focused on the *C. esculenta* flower may have hidden potential variations in richness in phenolic chemicals among other plant components (stem, rhizomes, and spadix). Using GC–MS/MS, this work discovered 20 bioactive secondary metabolites from ME‐CEF and assessed their biological potential, both in vivo and in vitro. The pertinent metabolites must be identified, described, and subjected to additional analysis to ascertain their mode of action, safety, efficacy, and dose profiles. This is followed by clinical trials to produce lead compounds from the extract. Toxicological characteristics, bioavailability, and pharmacokinetic/pharmacodynamic profiling of the plant extract also need to be investigated to produce new drugs. Through the identification of phytochemicals and the demonstration of significant bioactivities backed by in silico experiments, this work lays the groundwork for further investigations. 3,3′‐Thiodipropanol and *C. esculenta* exhibit antioxidant and hepatoprotective effects via Nrf2/ARE activation, Keap1‐Nrf2 interaction, NF‐κB modulation, PI3K/Akt, MAPK regulation, and glutathione metabolism.^58^, ^59^ 3,3′‐Thiodipropanol, an organosulfur compound with thioether (–S–) and hydroxyl (–OH) groups, exhibits antioxidant and hepatoprotective properties. It scavenges free radicals, modulates redox balance, and enhances antioxidant enzyme activity. By reducing lipid peroxidation and supporting detoxification pathways, it protects liver cells from oxidative damage, inflammation, and fibrosis. Its ability to counteract oxidative stress makes it a promising therapeutic agent for liver disorders, neurodegenerative diseases, and cardiovascular conditions. Further in vivo, in vitro, and pharmacokinetic studies are needed to confirm these mechanisms and optimize treatments for oxidative stress‐related liver diseases. Future research to fully utilize *C. esculenta*'s potential in a variety of applications will be guided by these investigations.

## 5 CONCLUSION

The results of our research indicate that the ME‐CEF has demonstrated significant promise in all areas of herbal therapy. Through in vitro, in vivo, and in silico investigations, our research has proven that the plant is rich in bioactive phytochemicals that have demonstrated antioxidant and hepatoprotective action.

Our research revealed that 3,3′‐thiodipropanol might be useful in treating low oxidation levels and providing a protective effect. However, additional study is needed to assess this chemical's efficacy as an antioxidant and hepatoprotective agent. Further investigation of *C. esculenta* flower components is essential for developing novel antioxidant and hepatoprotective drugs and understanding their mechanisms of action. To fully understand these drugs' therapeutic potential and provide patients with safe and effective treatment options, more research is necessary.

## AUTHOR CONTRIBUTIONS

**Mahathir Mohammad:** Formal analysis; investigation; methodology; writing – original draft; writing – review and editing. **Fahmida Tasnim Richi:** Conceptualization; formal analysis; investigation; methodology; visualization; writing – review and editing. **Rabiul Hossain:** Visualization; writing – review and editing. **Md. Arafat:** Validation; writing – review and editing. **Pair Ahmed Jiko:** Data curation; writing – review and editing. **Nazim Uddin Emon:** Conceptualization; formal analysis. **Sayed Al Hossain Rabbi:** Investigation; writing – review and editing. **Tirtha Khastagir:** Formal analysis; investigation. **Md. Hemayet Hossain:** Formal analysis; investigation; methodology. **Safaet Alam:** Conceptualization; methodology; project administration; supervision; validation; visualization.

## FUNDING INFORMATION

This work was partially funded by the Bangladesh Council of Scientific and Industrial Research (BCSIR) as an R&D project work of the 2022–2023 fiscal year, reference no.: 39.02.0000.011.14.157.2022/172 (Date: 10.11.2022).

## CONFLICT OF INTEREST STATEMENT

The authors declared no conflict of interest, which may influence the work reported in this manuscript.

## ETHICS STATEMENT

The Institutional Review Board of the University of Science & Technology, Chittagong, Bangladesh, approved this study (no.: USTC/EAC/24/020) for testing animal trials. The Swiss Academy of Science's criteria for managing animal models were followed, and established processes were followed when killing the animals.

## Data Availability

Data will be available upon valid request to the authors.
